# Anxiolytic effects of a galacto-oligosaccharides prebiotic in healthy females (18–25 years) with corresponding changes in gut bacterial composition

**DOI:** 10.1038/s41598-021-87865-w

**Published:** 2021-04-15

**Authors:** Nicola Johnstone, Chiara Milesi, Olivia Burn, Bartholomeus van den Bogert, Arjen Nauta, Kathryn Hart, Paul Sowden, Philip W. J. Burnet, Kathrin Cohen Kadosh

**Affiliations:** 1 School of Psychology, Faculty of Health and Medical Sciences, University of Surrey, Guildford, GU2 7XH UK; 2 BaseClear, Leiden, The Netherlands; 3 MyMicroZoo, Leiden, The Netherlands; 4 FrieslandCampina, Amersfoort, The Netherlands; 5 Department of Nutritional Sciences, School of Biosciences and Medicine, Faculty of Health and Medical Sciences, University of Surrey, Guildford, UK; 6 Department of Psychology, University of Winchester, Winchester, UK; 7 Department of Psychiatry, University of Oxford, Warneford Hospital, Oxford, UK

**Keywords:** Human behaviour, Microbiota, Anxiety

## Abstract

Current research implicates pre- and probiotic supplementation as a potential tool for improving symptomology in physical and mental ailments, which makes it an attractive concept for clinicians and consumers alike. Here we focus on the transitional period of late adolescence and early adulthood during which effective interventions, such as nutritional supplementation to influence the gut microbiota, have the potential to offset health-related costs in later life. We examined multiple indices of mood and well-being in 64 healthy females in a 4-week double blind, placebo controlled galacto-oligosaccharides (GOS) prebiotic supplement intervention and obtained stool samples at baseline and follow-up for gut microbiota sequencing and analyses. We report effects of the GOS intervention on self-reported high trait anxiety, attentional bias, and bacterial abundance, suggesting that dietary supplementation with a GOS prebiotic may improve indices of pre-clinical anxiety. Gut microbiota research has captured the imagination of the scientific and lay community alike, yet we are now at a stage where this early enthusiasm will need to be met with rigorous research in humans. Our work makes an important contribution to this effort by combining a psychobiotic intervention in a human sample with comprehensive behavioural and gut microbiota measures.

## Introduction

The gut microbiota has emerged as an important player in our efforts to understand the factors that influence brain function and behaviour^1–4^. The gut and the brain are intimately connected via the gut–brain axis, which involves bidirectional communication via neural, endocrine and immune pathways^5–7^. For example, gut microbial composition—which itself alters throughout the lifespan and in response to factors such as stress and lifestyle choices including diet^8^—has been shown to regulate gene expression and the release of metabolites in the brain^9,10^. There are also suggestions that a significant reduction in microbial diversity, or an increased number of pathogenic microbes, affects brain-behaviour relationships and may lead to psychological abnormalities that underlie mental illness^11,12^. To date, studies in humans have focused on characterising microbe populations in both health and disease^4,13^. In adults the gut microbiota has been related to atypical social functioning in autism^14^ and symptoms of anxiety and depression^7,15,16^. Animal research has suggested that the gut microbiota plays an important role during key moments in the host development, in particular during adolescence, which represents a critical time window where gut microbiota help fine-tune the gut–brain axis^2,8,17,18^. One consequence of gut microbiota variations during neurodevelopment is that it may lead to aberrant brain network maturation and thus atypical behavioural patterns. This supposition highlights the importance of understanding how changes in the gut microbiota relate to brain function and plasticity during this critical developmental period^18,19^.

Animal research has highlighted the significant impact of the gut microbiota on the development and maturation of brain networks that underlie emotional behaviour^20,21^. Particularly, nutritional interventions have been shown to fortify the gut–microbiota–brain axis and ameliorate microbial imbalances. Drastic changes in diet can alter microbial diversity in a matter of days^22^, and research also suggests that modifying microbial ecology via the intake of so-called ‘*psychobiotics*’ could help reduce stress responses and symptoms of anxiety and depression^23–25^. The term psychobiotics^3^ refers to live cultures of beneficial gut bacteria (*probiotics*) or substrates which enhance the growth and/or activity of indigenous beneficial intestinal bacteria (prebiotics), which, when ingested in sufficient amounts, improve brain function^26,27^. Probiotic strains, including members of the genera *Lactobacillus* and *Bifidobacterium,* are enriched in some dairy/fermented products, whereas prebiotics are non-digestible substances that feed the gut microbiota^22,28^ such as fructans and oligosaccharides found in cereals, fruits and vegetables^26^. Both pro- and prebiotics are commercially available and are applied in food products and supplements. The administration of psychobiotics (both probiotics and prebiotics) to rodents leads to robust, reproducible, attenuating effects on anxious and depressive-like behaviours, and suppress the neuroendocrine stress response. However, the translatability of these psychological effects to humans remains unclear.

A study in adult volunteers found that the consumption of probiotics in a randomised double-blind trial reduced measures of low mood and distress and urinary free cortisol which indicated a decreased stress level^29^. Similarly, a 4-week course of a multispecies probiotic in healthy participants resulted in reduced responsiveness to sad mood^30^. A neuroimaging investigation in female participants who had consumed a yoghurt containing probiotics over 4 weeks, revealed differential signals over brain regions involved in emotion processing and regulation^31^. Recent reviews and meta-analyses of such studies on probiotic effects for improving mental health outcomes have found modest effects in reducing depressive symptoms^32,33^, although there remains problematic between-study heterogeneity.

One potential drawback of using probiotics is that introducing an allochthonous probiotic species could disturb the cross-feeding microbiota population, which is particularly disruptive in participants with weakened immune systems^34^. In the current study, we therefore used a different approach with prebiotics to support the beneficial bacteria that are already present in the participants’ gut, such as *Bifidobacterium,* which have been linked to emotional well-being^29,30^.

Intake of a galacto-oligosaccharides (GOS) prebiotic over 3 weeks has also been shown to lower the secretion of the stress hormone cortisol and emotional processing in healthy adults^35^ in comparison to placebo. In the same study, participants exhibited decreased attentional vigilance to negative information in a dot-probe task. Given that anxious people routinely exhibit increased biases towards negative information^36^, this suggests that GOS intake may be useful in modifying anxiety-related psychological mechanisms. Presently, reviews and meta-analyses on the efficacy of prebiotics for reducing anxiety symptomology are mixed^33,37^, calling for further well controlled trials in human participants.

The main aim of the current study was to investigate whether GOS intake influences anxiety and mood measures in late adolescence and early adulthood in humans. Specifically, we used emotion regulation as a model for anxiety, as good emotion processing abilities in development are linked with various indices of well-being and mental health^38–40^. It has been repeatedly shown that the transitional period from adolescence to adulthood represents an important developmental juncture for both the emergence of social anxiety^38,39^ and the development of emotion control abilities, which allow the individual to control their fear responses and anxiety^41^. Given that adolescence also represents an important time point for fine-tuning the gut–brain axis^2^, our study adopted a two-pronged approach which would allow us to shed light on this unique transitional period into early adulthood from both a mental health and gut microbiota angle^42^. Here we compared the effects of a 4-week course of galacto-oligosaccharides (GOS) supplements compared to a placebo on the gut–microbiota and emotional behaviour and well-being. Our study aimed to replicate and extend the study by Schmidt et al. (2015) to a younger age range. Moreover, as in the original study, emotional behaviour was assessed with the attentional dot-probe task^35^ but we included a number of additional self-report measures of anxiety^43,44^, depression/mood^45,46^ and emotion regulation abilities^47,48^. In the current study we hypothesised that daily intake of GOS for 4 weeks would: (1) reduce self-reported levels of anxiety in the GOS group in comparison to the placebo group; (2) improve attentional bias towards positive emotional stimuli in the dot-probe task, and (3) stimulate the fecal abundance of potently beneficial gut bacteria (such as *Bifidobacterium*) in the GOS compared to the placebo group.

## Methods and materials

### Participants

Sixty-four healthy late adolescent female volunteers (aged 18–25 years) were recruited to this double-blind placebo-controlled 4-week galacto-oligosaccharides (GOS) supplement intervention study via posters and online advertisements. Inclusion criteria were no current or previous clinical diagnoses of anxiety or co-morbid neurological, psychiatric, gastrointestinal, or endocrine disorders; no current habitual use of prebiotic or probiotic supplements; no antibiotic use in the 3 months prior to study enrolment, no vegan diets and BMI ≤ 30 kg/m^2^. All participants provided written informed consent prior to testing and received financial compensation for participating in the study.

Participants were screened for trait anxiety scores on the STAI-Trait subscale 44 and using a custom programme blindly randomised to the supplement group as either ‘high’ or ‘low’ anxious by stratifying on the median of baseline trait scores for the recruited sample. This ensured an even distribution across the groups. Evaluating sample trait anxiety distribution found a small positive skew (0.28) indicating that broadly, sample mean (41.53) is equivalent to the median (40). Further, trait anxiety scores are similar to normative data for female undergraduate students (M = 38.25) 44. This study was approved by the University of Surrey Ethics Committee (UEC/2017/086/FHMS) and is registered on https://www.clinicaltrials.gov number NCT04616937 (registration date 05/11/2020). All testing and data processing was carried out in accordance with relevant guidelines and regulations.

### Sample size calculation

In replicating Schmidt and colleagues (2015) study, we determined our sample size based on the significant effects reported for GOS compared to placebo group in the dot probe task^35^. Here, we calculated the effect size to be small-medium (Cohen’s *d* = 0.27) and resultantly, estimated 48 participants to be required to detect significant effects in the dot probe task at 95% power and alpha level 0.05. Previous laboratory experience with the sample population suggests an attrition rate of approximately 20%. Thereafter, we specified recruitment of 60 participants.

### Design

At both testing time points, baseline and follow-up, the same testing protocol was used. All participants completed a comprehensive battery of self-report questionnaires (see supplementary materials description) assessing indices of anxiety^43,44^, mood^45,46^, emotion regulation^47,48^ and sleep^49^. Self-reported trait anxiety scores at baseline were used to group participants as high or low anxious based on the median score of the sample collected. Two subscales (verbal and matrix reasoning) of the Wechsler Abbreviated Scale of Intelligence^50^ were completed to estimate IQ, as well as the attentional dot-probe task to assess overt emotional processing^35^. Participants were provided with stool-sampling kits (MyMicroZoo, Leiden, The Netherlands) to self-collect faeces for gut microbiome sequencing analysis. Following baseline assessment, 28-day supply of supplements (GOS or placebo) were issued to be taken once daily. A 4-day food diary was completed at baseline and follow-up to assess usual nutrient intake at the start of the study and monitor compliance instructions to not change usual diet. Food diaries were reviewed by a member of the research team at testing appointments. Diaries were analysed using nutritional analysis software (https://www.nutritics.com/p/universityresearch) for energy and macro- and micronutrient intakes (supplementary information).

At follow-up, the entire testing protocol was repeated for each participant following cessation of supplement supply [study duration Mdn = 30 days for both GOS group (range 25–36 days) and placebo group (range 27–43 days)], and participants provided a second stool sample.

### Self-report measures

Participants completed a demographic questionnaire obtaining information on age, height, weight and relevant medical history. Following this, psychological self-assessment questionnaires obtained indices of state and trait anxiety (State-Trait Anxiety Inventory; STAI^44^), social anxiety (Social Anxiety Scale for Adolescents and Young People; SAS-A^43^), mood (Mood and Feelings Questionnaire: Short Version; MFQ^46^), and depression (Beck Depression Inventory-II; BDI-II^45^). Emotion regulation was indexed using the Emotion Regulation Questionnaire for Children and Adolescents; ERQ-CA^47^, and Thought Control Ability Questionnaire (TCAQ)^48^. Finally, participants reported sleep quality for the preceding 1-month period using the Pittsburgh Sleep Quality index (PSQI)^49^.

### Attentional dot-probe task

The attentional dot-probe task was adapted from Schmidt and colleagues^35^. Participants were presented with negative, positive and neutral word pairs to induce emotional bias, in both masked and unmasked conditions (to alter awareness of stimulus emotional valence). Experimental details can be found in the supplementary material). Attentional vigilance was calculated from the response times of correct responses by subtracting congruent RT from incongruent RTs for positive and negative stimuli in masked and unmasked conditions separately to give a bias score outcome for ‘positive bias’, where positive values indicate increased attention to emotional stimuli; and ‘negative bias’, where negative scores reduced attention to emotional stimuli.

### GOS/placebo supplement

Participants received either a daily dose of 7.5 g of the prebiotic galacto-oligosaccharides (Biotis™ GOS, ≈ 7.5 g powder ~ 5.5 g GOS) provided by FrieslandCampina Ingredients, Amersfoort, The Netherlands; or a placebo (maltodextrin, dried glucose syrup) for a period of 28 days. GOS are non-digestible carbohydrates, which are not completely broken down by human digestive enzymes. Because of this, they reach the intestine relatively intact, where they are then available for the present microbiota^27^ whereas maltodextrin is absorbed in the upper part of the intestine and does not reach the colon. Both supplements were provided in powdered form in unlabelled packaging and are similar in colour and taste. Supplements were instructed to be consumed by mixing with food or drink once daily.

### Stool sampling

At baseline and follow-up participants were provided with a unique sampling kit provided by MyMicroZoo (Leiden, the Netherlands) for stool collection at home. Feces samples were collected in DNA/RNA Shield (Zymo Research, CA, USA) and returned by the subjects to the recruitment station where collected samples were returned and stored at − 80 °C prior to being shipped on dry-ice for analysis by MyMicroZoo.

### DNA extraction

DNA extraction was performed using the Quick-DNA Fecal/Soil Microbe Miniprep Kit (Zymo Research) according to manufacturer’s instructions except for using the fecal slurry, containing DNA/RNA Shield, as input during bead beating for mechanical cell lysis instead of using the lysis buffer provided in the extraction kit.

### 16S rRNA gene based bacterial profiling

Illumina 16S rRNA gene amplicon libraries were generated and sequenced at BaseClear (Leiden, the Netherlands). See supplementary information for further details. Illumina reads were deposited in the European Nucleotide Archive (ENA) database (http://www.ebi.ac.uk/ena) in fastq-format under study accession number PRJEB32693 (or secondary accession number ERP115404). Abundance of each genera was calculated as a percentage of the total number of sequences identified in each sample. Shannon entropy of counts, a metric of microbiota diversity, was calculated using USEARCH 9.2^51^ with OTU clustering with a sequencing identity threshold of 97% after subsampling from the entire set, to account for different sampling depths.

### Gut microbiota differential abundance testing

The CoDaSeq R package^52,53^ was used to reduce genus-level microbiota reads (minimum 5000 with minimal proportion of 0.01 across all samples) to 86 taxa for differential analysis across intervention groups. Remaining zero reads were imputed using Bayesian-Multiplicative replacement of count zeros^54^ then standardised using the centre log-ratio transformation.

### Multivariate analysis

Partial Redundancy Analysis (RDA) was performed using the rda-function in the Vegan-package (version 2.5-4^55^) in R (version 3.3.1) to assess correlations between Hellinger transformed 16S rRNA gene based bacterial composition data (at the genus level) and the GOS treatment at baseline versus follow-up (as environmental variable) after conditioning the data for the covariate ‘subject’. RDA can be considered a constrained version of Principal Component Analysis (PCA). Where PCA considers all variance encompassed in the data, RDA only considers variance explained by the environmental variables (in this case time, contrasting baseline versus follow-up). The psychological response variables SAS, STAI Trait, STAI state, MFQ, BDI from the self-report measures, and positive and negative bias from attentional vigilance calculations in the dot-probe task were fitted onto the ordination. In the resulting biplot, a type 1 (object focused) scaling is employed so that the scores for samples are scaled by eigenvalues and that when plotted, the distances between them represent their similarity (Euclidean distances).

### Statistical analysis plan

#### A priori analysis

Prior to data collection, we planned to assess the influence of GOS on indices of anxiety and mood via self report measures and behavioural performance on the dot probe task and characterise GOS effects on the gut microbiota following 4 weeks intervention in comparison to a placebo group. Effects of supplement group on psychological and behavioural indices at follow up were assessed with analysis of covariance (ANCOVA) models on all self report measures and bias scores on the dot probe task, with baseline scores as a covariate. In reference to gut microbiota, Shannon diversity and differential abundance testing were assessed using the ANCOVA models. P-values resulting from the differential abundance tests were evaluated using Storey’s q-value^56^ to control for the positive false discovery rate. Multivariate analysis for gut bacterial composition, supplement group and psychological variables was assessed with Unrestricted Permutation Test.

#### Post hoc analysis

Given our interest in GOS effects on anxiety specifically we adapted our analyses to explore how trait anxiety levels influence outcomes. Our protocol for supplement group assignment and resultant normative distribution of self reported trait anxiety levels in our sample afforded the opportunity for us to stratify the supplement intervention groups by median trait anxiety score (as described under the section on participants) into high anxious (e.g. scores ≥ 40) or low anxious (e.g. scores < 40) groups to consider the effects and interactions of GOS and general anxiety levels. We focused on trait anxiety as this is characterised as a relatively stable feature enduring over a period of time rather than a transient and situational feeling as measured by the state subscale of the STAI (e.g. trait evaluation asks for ratings on ‘*how you generally feel*’ in comparison the state assessment which asks ‘*how you feel right now’*). Therefore, any changes associated with trait anxiety following supplement intervention may be indicative of sustained influence.

The additional anxiety factor was considered in reference to psychological and behavioural metrics (self-report measures, dot probe outcomes and in multivariate analysis considering correlations of these variables in reference to gut microbiota composition). Self-report measures and dot probe outcomes assessed by ANCOVA used anxiety group (high and low) as an additional factor. Multivariate analysis for gut microbiota composition, supplement group and psychological variables was also considered with anxiety grouping as an additional factor and tested with Unrestricted Permutation Test. Gut microbiota diversity and abundance assessment remained as described in a priori analysis. Note, we define *p-*values < 0.05 as significant, those between 0.05–0.10 trend-level significant and ≥ 0.10 non-significant. Statistical testing was performed in R version 3.5.1^57^.

## Results

A 25% attrition rate (see Supplementary Table 1 for an overview of attrite sub-sample) resulted in 48 participants completing the study, n = 23 in the GOS group (high anxiety n = 12, low anxiety n = 11) and n = 25 in the placebo group (high anxiety n = 13, low anxiety n = 12), similar in age [GOS median (Mdn) = 19 years (range 18–25), placebo Mdn = 20 years (range 18–25)], IQ [GOS Mdn = 103 (83–148), placebo Mdn = 102 (72–125)] and BMI [GOS Mdn = 20.5 kg/m^2^ (range 18.8–30.1), placebo = Mdn 20.3 kg/m^2^ (range 16.2–27.2)]. Diets were consistent across time for each supplement group for total energy consumption, although the GOS group had lower overall energy intake than placebo at both baseline (*p* = 0.009) and follow-up (*p* = 0.025). At the macronutrient level, consumption was estimated as a percentage of energy intake for each individual over the first (baseline) and last (follow-up) 4-day period of supplement intake. Between GOS and placebo groups, non-parametric tests showed no significant difference for carbohydrate, fat, protein, dietary saturated fat, fibre, sugars or alcohol intake. From baseline to follow-up paired sample non-parametric tests showed no difference in any of the aforementioned measures in the placebo group, but the GOS group demonstrated a reduction in sugar consumption at follow-up (*p* = 0.010), with all other measures remaining consistent (Supplementary Table 2).

### A priori analysis

Models for self report measures, attentional dot-probe outcomes, and multivariate analysis found no evident effects in any measure. We therefore implemented and report on our post hoc analyses for these tests.

### Intervention effects on gut microbiota

Descriptive means of Shannon diversity are displayed in Supplementary Table 5. There was a trend level increase in Shannon diversity (*F*(1,43) = 3.76 *p* = 0.059, n^2^ = 0.027), a small effect driven by a larger Shannon index in the placebo group compared to the GOS group, suggesting taxa abundances at follow up were more equally represented in the placebo group. Differential abundance testing of 86 taxa found no initial baseline differences in supplement groups thus ANCOVA models were applied assessing intervention effects at follow up. Eight taxa (Table 1) found p-values < 0.1, 2 of which were < 0.05 indicating differential abundances between placebo and GOS intervention. After controlling for positive false discovery rate with Storey’s q-value, our smallest sample q-value was 0.548 represented by 21 taxa, with a false discovery rate of 50–53%, implying that approximately half of these are false positives. Of these, 8 presented with a p-value < 0.10, and are all displayed in Table 1. The taxa *Bifidobacterium* features with a 0.80% increase in growth in the GOS group that is absent in the placebo group (0.05% reduction) and is also 0.74% more abundant following intervention in the GOS group compared to placebo, signifying a medium intervention effect.

**Table 1:** Sample taxa resulting from differential abundance testing of intervention effects.

Class	Order	Family	Genus	Change from T1 to T2		Difference [ref placebo]		
				GOS	Placebo	T2	η^2^	Effect
**Phylum: Bacteroidetes**								
Bacteroidia	Bacteroidales	Bacteroidaceae	*Bacteroides*	0.20% ↓	0.20% ↑	0.17% ↑	0.025	Small
Bacteroidia	Bacteroidales	Porphyromonadaceae	*Barnesiella*	0.59% ↑	0.36% ↓	0.59% ↑	0.039	Small
**Phylum: Actinobacteria**								
Actinobacteria	Bifidobacteriales	Bifidobacteriaceae	*Gardnerella*	0.18% ↑	0.71% ↑	0.62% ↓	0.044	Medium
Actinobacteria	Bifidobacteriales	Bifidobacteriaceae	*Bifidobacterium*	0.80% ↑	0.05% ↓	0.74% ↑	0.041	Medium
**Phylum: Proteobacteria**								
Alphaproteobacteria	Rhodospirillales	Rhodospirillaceae	*Aestuariispira*	0.53% ↑	0.31% ↓	0.84% ↑	0.052	Medium
Deltaproteobacteria	Desulfovibrionales	Desulfovibrionaceae	*Desulfovibrio*	0.15% ↑	0.55% ↓	0.97% ↑	0.026	Small
**Phylum: Firmicutes**								
Tissierellia	Tissierellales	Peptoniphilaceae	*Peptoniphilus*	0.03% ↓	0.66% ↑	0.78% ↓	0.067	Medium
Clostridia	Clostridiales	Ruminococcaceae	*Sporobacter*	0.36% ↑	0.18% ↓	0.17% ↑	0.019	Small

Differential taxa identified at follow up (T2) from ANCOVA models. Descriptive changes in abundance from baseline (T1) measures to follow up are displayed for each intervention group and effect size of abundance group difference at T2. Upward arrows represent an increased/more abundance, and downward arrows decreased/less abundance taxa.

### Post hoc analysis

#### Self-report measures

For all measures, models comparing baseline scores confirmed no differences between groups thus ANCOVA was used to compare group means at follow-up (Supplementary Table 3). The significant interaction of intervention and anxiety grouping on trait anxiety scores (*F*(1,42) = 5.58, *p* = 0.023, n^2^ = 0.03) was followed up with further ANCOVAs examining the influence of intervention for anxiety grouping independently. It was found that, compared to the placebo group, 4 weeks of GOS consumption reduced self-reported scores for high anxious participants at trend-level (*F*(1,21) = 3.88, *p* = 0.062, n^2^ = 0.12; adjusted means GOS *M* = 45.47, *SE* = 1.43 [CI 42.49–48.43]; placebo *M* = 49.45, *SE* = 1.43 [CI 46.48–52.42]) but not for the low anxious group (*F*(1,20) = 1.84, *p* = 0.190, GOS *M* = 32.34, *SE* = 1.59 [CI 29.03–35.65]; Placebo *M* = 29.36, *SE* = 1.52 [CI 26.19–32.52]) (Supplementary Figure 1). There were no other interactions between intervention group and anxiety group for social or state anxiety, or in mood measures (BDI or MFQ), emotion regulation or sleep quality index (Supplementary Table 4).

#### Attentional Dot-probe task

**Figure 1:**
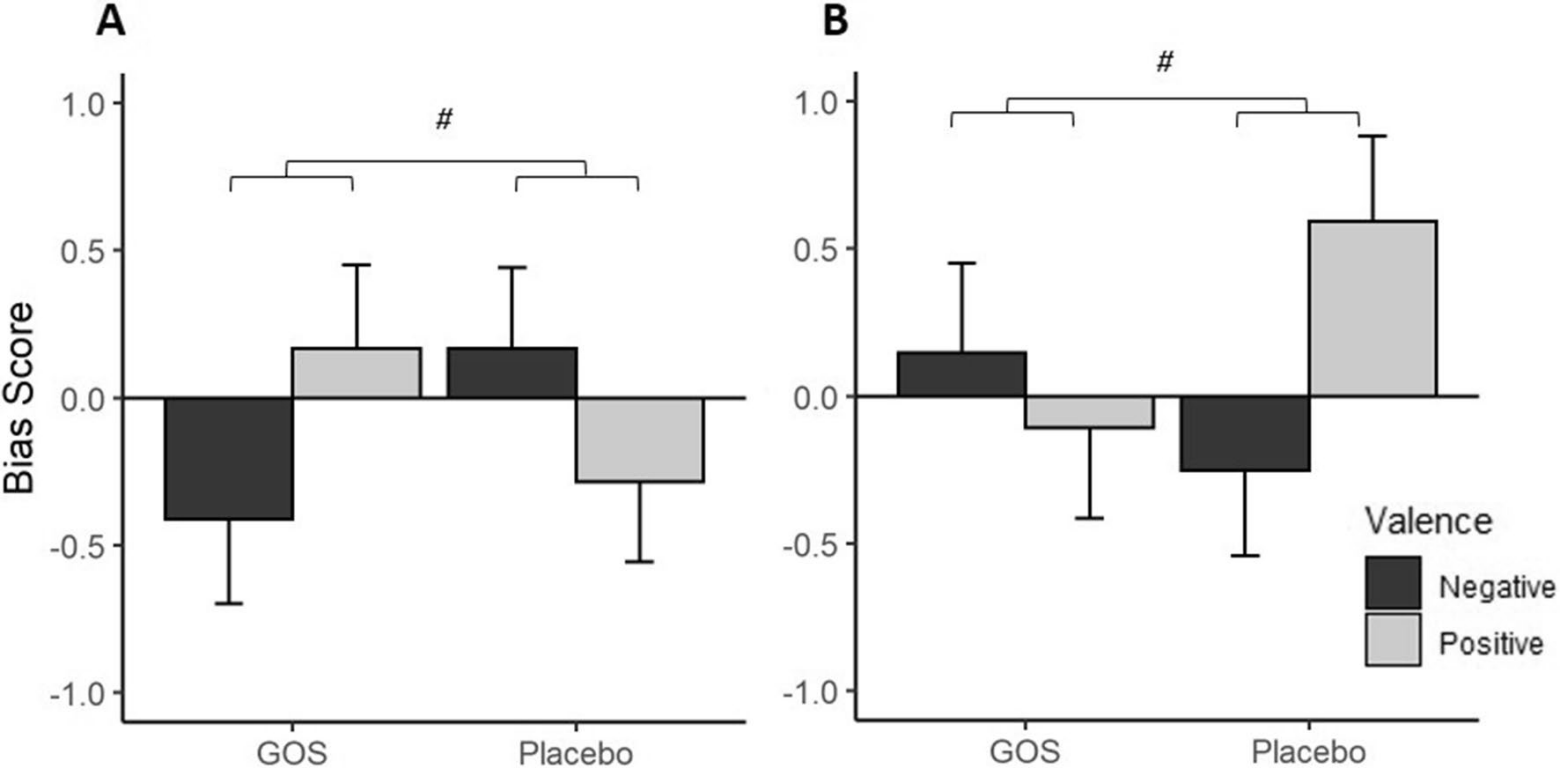
Interaction of attentional vigilance to stimulus valence (y-axis, bias score (z-scores)) by intervention group in the high anxious group (**A**), and the low anxious group (**B**) at follow-up. Error bars illustrate SE (**A**), high anxious GOS group shows a trend towards reduced bias to negative stimuli, and increased bias to positive stimuli in comparison to the placebo group. *p = 0.070. (**B**) Low anxious Placebo group shows a trend towards reduced bias to negative stimuli, and increased bias to positive stimuli in comparison to the GOS group. *p = 0.069.

ANCOVA models were applied to dot probe outcomes after confirming no baseline differences between intervention groups. For the factors block (masked and unmasked), emotional valence (positive and negative), intervention group (GOS and placebo) and anxiety (high and low) a 4-way interaction was found; (*F*(1,175) = 4.26, *p* = 0.04, n^2^ = 0.022). To investigate where the significant difference lay, this interaction was modelled by the block factor in separate ANCOVAs for masked and unmasked conditions. There was no significant interaction in the masked block, (*F*(1,87) = 0.07, *p* = 0.788) however, there was a significant interaction with emotional valence, intervention group and anxiety group in the unmasked block (*F*(1,87) = 7.10, *p* = 0.009, n^2^ = 0.073). This was further modelled by anxiety grouping independently to examine the influence of intervention group on emotional valance stimuli (Fig. 1). In the high anxious group, there was a trend towards an interaction between intervention group and valence condition (*F*(1,45) = 3.46, *p* = 0.070, n^2^ = 0.071) where participants in the GOS group in comparison to the placebo group showed reduced bias to negative stimuli (GOS *M* = − 34.86 ms, *SE* = 18.86 [CI − 72.86 to 3.13]; Placebo *M* = 3.67 ms, *SE* = 18.18 [CI − 32.95 to 40.29]) and greater bias to positive stimuli, (GOS *M* = 3.62 ms, *SE* = 18.90 [CI − 34.45 to 41.69]; Placebo *M* = − 26.36 ms, *SE* = 18.09 [CI − 62.82 to 10.08]). The trend towards interaction between intervention group and valence condition was also found in the low anxious group (*F*(1,41) = 3.49, *p* = 0.069, n^2^ = 0.073). Unlike in the high anxious participants, the low anxious placebo group in comparison to the GOS group demonstrated decreased bias to negative stimuli (GOS *M* = 2.28 ms, *SE* = 20.07 [CI − 38.26 to 42.83]; Placebo *M* = -24.15 ms, *SE* = 19.29 [CI − 63.11 to 14.80]) and increased bias to positive stimuli (GOS *M* = − 14.63 ms, *SE* = 20.34 [CI − 55.73 to 26.45]; Placebo *M* = 31.81 ms, *SE* = 19.33 [CI − 63.11 to 14.80]).

#### Multivariate analysis

**Figure 2:**
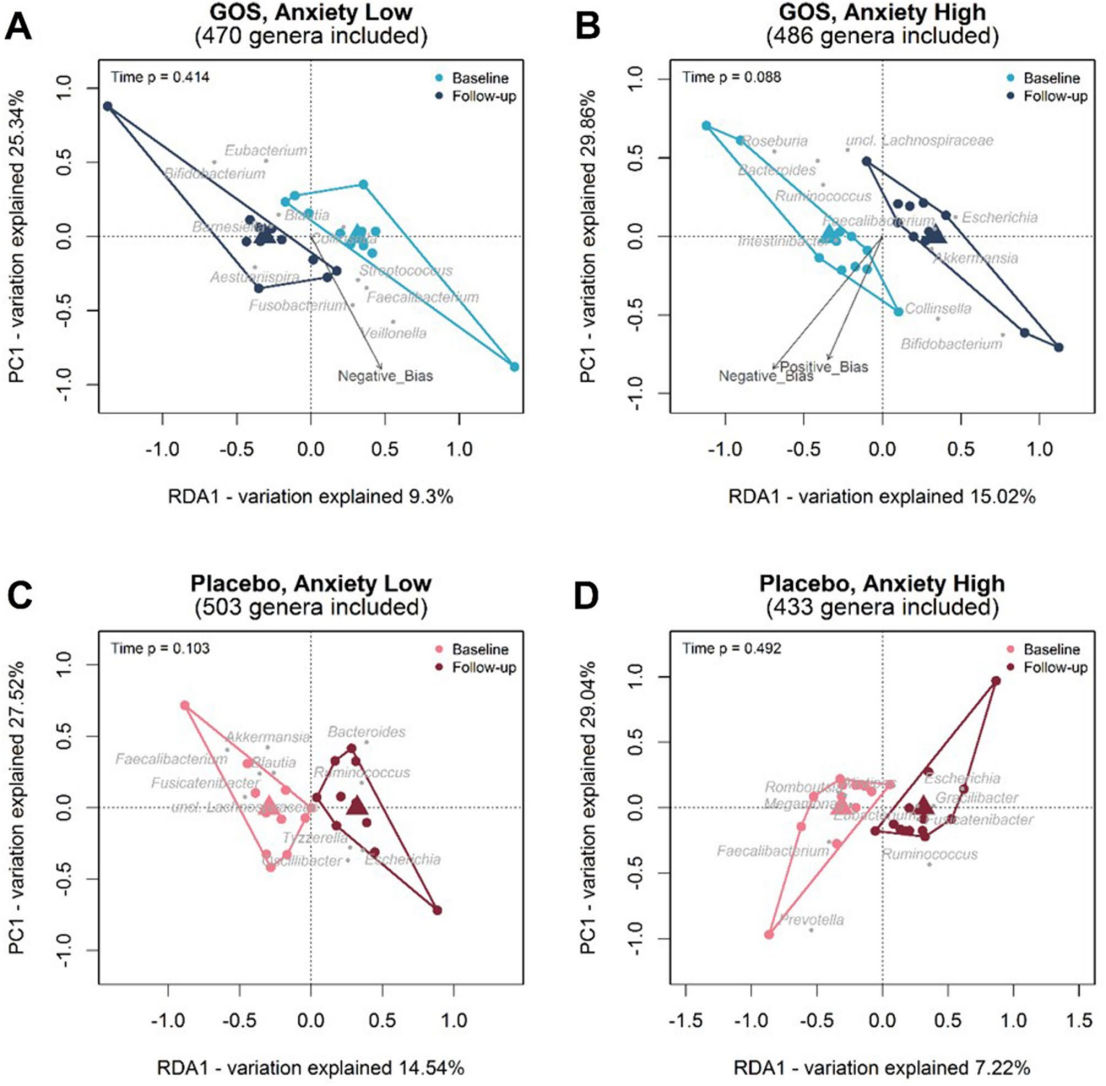
Ordination diagram from RDA for samples from the placebo and GOS group collected at baseline and follow-up, where the RDA indicates the association between Time [explanatory variables (x axis)] and bacterial community data on the Genus level. Scores of the first RDA-axis are plotted on the x-axis and scores of the first PCA-axis are plotted on the y-axis. Individual samples are represented by points that are coloured by time and samples belonging to the same collection time are enveloped. Triangles in the RDA diagrams represent the class centroids. Grey points and labels represent the 10 best-fitting genus level bacterial groups. The psychological measurements (environmental variables) are fitted onto the ordination and indicated by the named arrows. The arrow shows the direction of the (increasing) gradient, and the length of the arrow is proportional to the correlation between the variable and the ordination. Only those psychological measurements with a p value of < 0.10 are added to the ordination. The calculated p-value from Unrestricted Permutation Test is added to the upper left corner of the RDA diagram.

RDA analysis was performed for both the GOS and placebo group and anxiety group separately contrasting measures across time from baseline to follow-up. Results are displayed in the ordination diagrams in Fig. 2. For individuals in the GOS group classified as high anxious at baseline, genus level gut microbiota communities showed a trend level difference at follow-up (p = 0.088). For the GOS low anxious group, there was no significant prediction of gut microbiota genera on psychological measures across time (*p* = 0.414). In the placebo groups, the low anxious participants illustrate no differences between microbiota communities from baseline to follow-up (*p* = 0.103), similar to the high anxious participants (*p* = 0.492).

## Discussion

In this double-blind placebo-controlled 4-week galacto-oligosaccharides (GOS) supplement intervention study we sought to characterise the influence of GOS on indices of emotional well-being and microbial gut composition in a sample of female participants towards the end of adolescence and into early adulthood. We found anxiolytic effects of GOS in high anxious participants in self-reported trait anxiety and trends towards reduced negative emotional bias and increased positive bias in the dot-probe task. Additionally, gut microbiota composition was characterised by increased *Bifidobacterium* abundances at follow-up in the GOS group, with trends towards differential diversity after intervention. Multivariate analysis of microbial composition against psychological measures found trend level separation of bacterial composition in high anxious females at follow-up compared to baseline. Altogether, these data indicate that GOS supplementation has an anxiolytic influence on emotional wellbeing in high anxious late adolescent females, complemented by changes in gut microbiota composition.

The anxiolytic effects evident in this intervention are in line with a prior community study of GOS whereby stress indicators were reduced and emotional behaviour improved following 3 weeks of GOS supplementation^35^, adding corroborative evidence to the literature that GOS supplementation assists in functional enhancement of biological networks underpinning emotion regulation and mood^58^. Anxiolytic and antidepressant effects of multispecies pre- and probiotics are apparent in several in-human studies e.g.^29–31,35,59^, however, results of this study showed no demonstrable impact of GOS on measures of mood, depression, emotion regulation or indeed social or state reported anxiety levels at the group level. Here, only the high anxious prebiotics group reported a significant reduction in trait anxiety. Some *clinical* trials have linked pro- or prebiotic intake to reduced depression^30^ or anxiety^60,61^, others have found no support^62–65^. In these trials, measures of depression and anxiety are usually secondary to primary outcomes in overall improvement in clinical conditions^66^ and are comorbid in the function of existing dysregulated biological systems (e.g. IBS^60,61^, rheumatoid arthritis^66^, fibromyalgia^63^). The finding that GOS only impacts self-reported sub-clinical trait anxiety in this study, in the absence of a significant differential effect of supplement group and anxiety level for self-reported state anxiety or depression measures is surprising (note though that a significant reduction in state anxiety level was found across all groups) and suggests a dimension of sensitivity of GOS intake on general anxiety levels in young adult females that would benefit from further exploration.

Trait anxiety is a persistent emotional state characterised by doubts, fear and worry. When such a state co-occurs with the final period of maturation in late adolescents, emotion regulation ability is key for determining the trajectory for lifelong behaviours^39^. Animal models have shown that dysbiosis in adolescence results in lasting effects on brain–behaviour interactions^8,17,67^. While microbiota–gut–brain (MGB) influences are bidirectional, biological systems may be fine-tuned via nutritional intake. This may benefit late adolescent females transitioning into young adulthood, especially those subject to transient suboptimal emotional regulation skills in the final stages of maturation (e.g. displaying symptoms of stress and anxiety). Nutritional supplementation and consequential influence on MGB may prove effective in stabilising some of these symptoms in this age group. Probiotics are known to influence the gut–brain axis via endocrine, immune and neural pathways^31,68,69^. One such pathway from the gut microbiota on the brain is via the creation of short-chain fatty acids (SCFAs) by bacterial fermentation in the gut^70^, although most evidence to date is based on animal research. In human participants, it has been shown that *Lactobacillus rhamnosus* intake exerts influence on GABA receptors in the brain^68^, which cooperates with excitatory glutamate to regulate brain function. Glutamate and GABA are respectively excitatory and inhibitory neurotransmitters that are essential in typical cognitive development^71^. In anxious individuals, disruption to the balance of this relationship is correlated with poorer cognitive control^72^ and influences emotion regulation ability. The intake of pre- and probiotics may contribute to the harmonisation of the excitatory/inhibitory balance via the gut–brain axis. This is an exciting area of research in which functional gut microbiota can influence host behaviour via physiological processes, and may offer biological solutions to alter the trajectory of psychological problems.

We affirmed a clear influence of GOS intake on *Bifidobacterium* increase across time in comparison to placebo group, adding to the evidence that *Bifidobacterium* bacteria may be a driver of improving mental well-being. We also found greater diversity in the placebo group compared to the GOS group, it may be that promoting the growth of prebiotic bacteria could have adaptive effects in preventing the growth of less beneficial bacteria. However, it is difficult to disentangle if the influence of GOS observed in these data is due to the stimulation of *Bifidobacterium* growth, or a synthetic reduction.

Another possible explanation relates stress to microbiota volatility^73^, Bastiaanssen and colleagues reliably illustrated that microbiota instability is linked to increased stressors over time. This suggest that changes in microbial diversity may indicate greater physiological stress via neuroendocrine feedback loops. We found greater diversity in the placebo group compared to the GOS group. We speculate this may reflect a greater degree of stress related volatility in the placebo group that was absent if GOS supplementation prevented stress-related alterations, here evidenced by anxiolytic effects, supported by RDA analysis which found trend level separation of gut microbiota composition in high anxious participants. This makes stress-related volatility an interesting concept worth exploring in future studies. Naturally, diet is likely a key component of this relationship and in this sample, we found little support for dietary changes in groups across time, excepting reduced sugar intake in the GOS group, or between groups. This strengthens the hypothesis that GOS exerts beneficial influence on well-being, yet the biological mechanisms require further investigation. Regardless, the subtle fine tuning of microbiota with GOS intake may be enough to assist in the biological regulation of emotional pathways and contribute to improved well-being in pre-clinical populations.

Of note in the outcome of this study is the relatively small effect sizes and trend level p-values of our self-report psychological measures and dot-probe task. The dot-probe task encapsulates attentional biases to emotional stimuli. Prevalent, heightened anxiety commonly results in attentional bias to negative, or threating information^36^, and can be reduced by attentional bias modification training^74^. Attentional training works to modify emotion regulation networks in the brain^75^, however, there are questions surrounding the consistency of the manifestation of attentional biases at the individual level^75^, and in particular at the pre-clinical level^76^. In the absence of attentional bias training, results from the dot-probe task were indicative of trends towards reduced negative bias and increased positive bias to emotional stimuli in the high anxious GOS group, a pattern that was also observed in the low-anxious placebo group. While this may indicate that high anxious GOS participants have undergone alterations in cognitive-emotional processing compared to the high anxious placebo group, it is difficult to cleanly interpret as the low anxious GOS group results do not mirror their placebo counterparts. Although there are trends towards interventional effects, individual variability in anxiety expression within intervention groups may be influenced by variables not accounted for in this study. One such factor could be the use of hormonal contraceptives, which have been shown to affect emotion regulation and mood^77^.

We note that similar community sample research has produced equivalent small-medium effect sizes on comparative measures^35^. This might be anticipated in an intervention in sub-clinical typical populations examining factors at group level where specific pathways and mechanisms are yet to be fully established, however there is a methodological aspect to consider in this study. Our initial design was powered for comparing two supplement groups across time where as our post hoc analyses required an additional factor (anxiety levels) thus reducing power to detect effects. Power achieved for effects seen in the dot-probe task was 54%, trait anxiety 65% and *Bifidobacterium* difference 44% and this should be carefully considered while interpreting these results. While power > 80% is generally considered sufficient to avoid both the detection of false positive results and the rejection of false negatives (Type I and II errors) our results fall below this. However, considering prior research in humans and animals as discussed, we suggest that these data provide informative contributions regarding emotional processes and gut composition influenced by GOS supplementation to the existing literature, particularly in a sub-clinical anxious population.

Further research is required with improved power considering the small-medium effects identified here. In addition, well defined operational measures (e.g. behavioural indices of anxiety) with strong correlations to mediating pathways as exhibited in animal studies may result in more targeted therapeutic potential of GOS in humans. Herein, GOS has been established to increase *Bifidobacterium* abundance in 4 weeks in co-occurrence with reduced anxiety manifestation, with indications of modification of attentional bias in a sample of adolescent females. Data presented here are indicative that GOS may be effective in influencing the expression of anxiety and would benefit from further research specifying potential pathways of this effect.

## Supplementary Information

Supplementary Information.

## Data Availability

Data and code for reproducible analysis are available at 10.17605/OSF.IO/NGMSU.
