# Supplementary material for: Anxiolytic effects of a galacto-oligosaccharides prebiotic in healthy females (18–25 years) with corresponding changes in gut bacterial composition
Source: Sci Rep. 2021 Apr 15;11:8302. doi: 10.1038/s41598-021-87865-w (PMC8050281; doi:10.1038/s41598-021-87865-w)
Supplement: Supplementary file 1 — Supplementary Information. [file 41598_2021_87865_MOESM1_ESM.docx]

**Anxiolytic effects of a galacto**-**oligosaccharides prebiotic in healthy females (18-25 years) with corresponding changes in gut bacterial composition**

Nicola Johnstone^1^, Chiara Milesi^1^, Olivia Burn^1^, Bartholomeus van den Bogert^2,3^, Arjen Nauta^4^ , Kathryn Hart^5^, Paul Sowden^1,6^, Philip WJ Burnet^7^, & Kathrin Cohen Kadosh^1^

*^1^School of Psychology, Faculty of Health and Medical Sciences, University of Surrey, Guildford, UK*

*^2^BaseClear, Leiden, The Netherlands*

*^3^MyMicroZoo, Leiden, The Netherlands*

*^4^FrieslandCampina, Amersfoort, The Netherlands*

*^5^Department of Nutritional Sciences, School of Biosciences and Medicine, Faculty of Health and Medical Sciences, University of Surrey, Guildford, UK*

*^6^Department of Psychology, University of Winchester, Winchester, UK*

*^7^Department of Psychiatry, University of Oxford, Warneford Hospital, Oxford, UK*

**Corresponding authors:**

*Kathrin Cohen Kadosh*, School of Psychology, Faculty of Health and Medical

Sciences,

University of Surrey, Guildford, GU2 7XH. Phone: +44(0) 1483 68 3968. Email:

[k.cohenkadosh@surrey.ac.uk](mailto:k.cohenkadosh@surrey.ac.uk) URL: kcohenkadosh.com

*Nicola Johnstone*, School of Psychology, Faculty of Health and Medical Sciences, University of Surrey, Guildford, GU2 7XH. Phone: +44(0) 1483 68 4195. Email: [nicola.johnstone@surrey.ac.uk](mailto:nicola.johnstone@surrey.ac.uk)

**Supplementary Information**

**Methods and Materials**

*Food Diaries.* The food diary is a semi-quantitative diary used routinely by our group, which required participants to record their intake concurrently during the day in as much detail as possible, including brand names, cooking methods and portion sizes.

*Psychological self-report measures.* Participants completed a demographic questionnaire obtaining information on age, height, weight and relevant medical history. Following this, psychological self-assessment questionnaires obtained indices of state and trait anxiety (State-Trait Anxiety Inventory; STAI ^1^), social anxiety (Social Anxiety Scale for Adolescents and Young People; SAS-A ^2^), mood (Mood and Feelings Questionnaire: Short Version; MFQ ^3^), and depression (Beck Depression Inventory-II; BDI-II ^4^). Emotion regulation was indexed using the Emotion Regulation Questionnaire for Children and Adolescents; ERQ-CA, ^5^, and Thought Control Ability Questionnaire (TCAQ), ^6^. Finally, participants reported sleep quality for the preceding 1-month period using the Pittsburgh Sleep Quality index (PSQI), ^7^.

*Attentional dot probe task.* This task was included to measure emotional bias behaviourally, using emotional words (positive, negative and neutral) in both masked and unmasked conditions to alter awareness of stimulus emotional valence. Participants were positioned approximately 1 meter from a 19” LED monitor (native resolution 1280 x 1024 SXGA, brightness 250 cd/m^2^) on which white stimuli were presented (size 36 Aerial font) on a black background. E-Prime 2.0 (Psychology Software Tools, Pittsburgh, PA) was used to present experimental stimuli, and responses collected by keyboard with 1000Hz polling rate. At the beginning of each trial, a fixation cross appeared on screen, followed with a word pair that was one of 30 positive-neutral, 30 negative-neutral and 30 neutral-neutral pairs, with words positioned at the top and bottom of the screen. Emotional word position was equally split across top and bottom. In the masked condition (90 trials), word pairs were presented for 17 ms, followed by a length and position matched mask of a nonsense letter string for 483 ms. In the unmasked condition (90 trials), word pairs were presented for 500 ms. This was followed by a probe of either one or two stars, that was congruent or incongruent with the emotional word position. The participant responded by pressing either ‘1’ or ‘2’ corresponding to the number of stars in the probe, terminating the trial. Frequency of number of stars and of position was equally split across all trials. A total of 180 trials were presented and randomised across masked and unmasked conditions, and valence stimulus pairs. Attentional vigilance was calculated from the response times of correct responses by subtracting congruent RT from incongruent RTs for positive and negative stimuli in masked and unmasked conditions separately.

*16S rRNA gene based bacterial profiling*. Barcoded amplicons from the V3-V4 region of 16S rRNA genes were generated using a 2-step PCR. 10 genomic (g)DNA was used as template for the first PCR with a total volume of 50 ul using the 341F (5’-CCTACGGGNGGCWGCAG-3’) and the 785R (5’-GACTACHVGGGTATCTAATCC-3’) primers appended with Illumina adaptor sequences. PCR products were purified, and the size of the PCR products were checked on Fragmentanalyzer (Advanced Analytical, CA, USA) and quantified by fluorometric analysis. Purified PCR products were used for the 2nd PCR in combination with sample-specific barcoded primers (Nextera XT index kit; Illumina, CA, Illumina). Subsequently, PCR products were purified, checked on a Fragment analyser and quantified, followed by multiplexing, clustering, and sequencing on an Illumina MiSeq with the paired-end (2x) 300 bp protocol and indexing. The sequencing run was analysed with the Illumina CASAVA pipeline (v1.8.3) with demultiplexing based on sample-specific barcodes. The raw sequencing data produced was processed removing the sequence reads of too low quality (only "passing filter" reads were selected) and discarding reads containing adaptor sequences or PhiX control with an in-house filtering protocol. A quality assessment on the remaining reads was performed using the FASTQC quality control tool version 0.10.0. The Illumina paired reads were merged into single reads (so-called pseudoreads) through sequence overlap, after removal of the forward and reverse primers. Chimeric pseudoreads were removed using USEARCH 9.2 ^8^ and the remaining reads were aligned to the RDP 16S rRNA gene database ^9^. Based on the alignment scores of the pseudoreads, the taxonomic depth of the lineage is based on the identity threshold of the rank; Species 99%, Genus 97%, Family 95%, Order 90%, Class 85%, Phylum 80%.

**Results**

**Supplementary Table 1**. Attrition sub-sample overview of psychological measures

| Supplement Group | Anxiety group | *n* | Age (*mdn* years) | STAI  Trait | STAI  State | SAS | BDI | MFQ | ERQ Re-appraisal | ERQ  Suppression |
| --- | --- | --- | --- | --- | --- | --- | --- | --- | --- | --- |
| GOS | High | 3 | 20 | 52 | 33 | 51 | 19 | 11 | 20 | 13 |
|  | Low | 8 | 19 | 31 | 28 | 39 | 4 | 2 | 18 | 8 |
| Placebo | High | 4 | 22 | 60 | 58 | 50 | 19 | 16 | 22 | 14 |
|  | Low | 1 | 20 | 31 | 25 | 22 | 15 | 5 | 25 | 8 |

*Note.* Psychological questionnaire scores are mean values.

**Supplementary Table 2.** Means and non-parametric test significance values of food diary entries for the first and final four days of supplement intake.

| **Macro as % of total energy** |  | **Baseline** | **Follow Up** | **Within (time)*p*** | | |
| --- | --- | --- | --- | --- | --- | --- |
|  |  | *Mean* (*SD*) | *Mean* (*SD*) |  | | |
| *Energy intake* (Total, Kcal) |  |  |  |  | | |
| GOS |  | 1631.67 (338.08) | 1654.63 (393.85) | >0.999 | | |
| Placebo |  | 1921.61 (418.32) | 1905.29 (461.38) | 0.353 | | |
|  | **Between (group) *p*** | **0.009** | 0.025 |  | | |
| *Carbohydrates* |  |  |  |  | | |
| GOS |  | 45.28 (7.12) | 45.66 (7.09) | 0.402 | | |
| Placebo |  | 47.19 (6.41) | 47.83 (7.15) | 0.498 | | |
|  | **Between (group) *p*** | 0.536 | 0.416 |  | |  |
| *Fat* |  |  |  |  | | |
| GOS |  | 36.05 (6.54) | 35.37 (5.73) | 0.675 | | |
| Placebo |  | 35.01 (6.04) | 34.68 (6.21) | 0.800 | | |
|  | **Between (group) *p*** | 0.490 | 0.475 |  | | |
| *Protein* |  |  |  |  | | |
| GOS |  | 16.18 (4.54) | 16.08 (4.46) | 0.529 | | |
| Placebo |  | 15.35 (4.33) | 14.90 (4.17) | 0.076 | | |
|  | **Between (group) *p*** | 0.703 | 0.575 |  | | |
| *Dietary Saturated Fat* | | |  | |  | |
| GOS |  | 13.53 (6.03) | 13.10 (5.98) | 0.834 | | |
| Placebo |  | 11.64 (3.12) | 11.64 (3.08) | 0.673 | | |
|  | **Between (group) *p*** | 0.381 | 0.489 |  | | |
| *Sugars* |  |  |  |  | | |
| GOS |  | 18.85 (7.61) | 16.24 (4.92) | **0.010** | | |
| Placebo |  | 18.95 (6.79) | 19.27 (8.21) | 0.715 | | |
|  | **Between (group) *p*** | 0.942 | 0.287 |  | | |
| *Alcohol* |  |  |  |  | | |
| GOS |  | 2.35 (4.15) | 2.73 (4.32) | 0.418 | | |
| Placebo |  | 2.44 (3.90) | 2.60 (3.97) | >0.999 | | |
|  | **Between (group) *p*** | 0.965 | 0.972 |  | | |
| **Fibre (g)** |  |  |  |  | | |
| GOS |  | 18.36 (6.14) | 18.18 (6.96) | 0.529 | | |
| Placebo |  | 18.56 (7.11) | 18.83 (7.21) | >0.554 | | |
|  | **Between (group) *p*** | 0.910 | 0.758 |  | | |

**Supplementary Table 3.** ANCOVA interaction for SRMs

|  | term | df | F | p.value | etasq | power |
| --- | --- | --- | --- | --- | --- | --- |
| *Anxiety Measures* | |  |  |  |  |  |
| **Trait** | Baseline | 1 | 129.89 | 0 | 0.688 | 1.00 |
|  | Group | 1 | 0.14 | 0.707 | 0.001 | 0.07 |
|  | Anxiety | 1 | 11.15 | 0.002 | 0.059 | 0.92 |
|  | Intervention:Anxiety | 1 | **5.58** | **0.023** | **0.03** | **0.66** |
|  | Residuals | 42 |  |  |  |  |
| **State** | Baseline | 1 | 32.84 | 0 | 0.396 | 1.00 |
|  | Group | 1 | 0.31 | 0.581 | 0.004 | 0.09 |
|  | Anxiety | 1 | 4.88 | 0.033 | 0.059 | 0.60 |
|  | Intervention:Anxiety | 1 | 1.85 | 0.181 | 0.022 | 0.27 |
|  | Residuals | 43 |  |  |  |  |
| **SAS-A** | Baseline | 1 | 171.70 | 0 | 0.795 | 1.00 |
|  | Group | 1 | 0.03 | 0.859 | 0 | 0.05 |
|  | Anxiety | 1 | 0.34 | 0.561 | 0.002 | 0.09 |
|  | Intervention:Anxiety | 1 | 0.80 | 0.377 | 0.004 | 0.15 |
|  | Residuals | 43 |  |  |  |  |
| *Mood Measures* | |  |  |  |  |  |
| **BDI** | Baseline | 1 | 65.62 | 0 | 0.575 | 1.00 |
|  | Group | 1 | 1.80 | 0.186 | 0.016 | 0.27 |
|  | Anxiety | 1 | 2.79 | 0.102 | 0.024 | 0.39 |
|  | Intervention:Anxiety | 1 | 0.88 | 0.354 | 0.008 | 0.16 |
|  | Residuals | 43 |  |  |  |  |
| **MFQ** | Baseline | 1 | 26.83 | 0 | 0.354 | 1.00 |
|  | Group | 1 | 0.25 | 0.622 | 0.003 | 0.08 |
|  | Anxiety | 1 | 5.22 | 0.027 | 0.069 | 0.63 |
|  | Intervention:Anxiety | 1 | 0.52 | 0.475 | 0.007 | 0.11 |
|  | Residuals | 43 |  |  |  |  |
| *Thought Regulation* | |  |  |  |  |  |
| **Reappraisal** | Baseline | 1 | 23.74 | 0 | 0.335 | 1.00 |
|  | Group | 1 | 0.26 | 0.616 | 0.004 | 0.08 |
|  | Anxiety | 1 | 2.55 | 0.118 | 0.036 | 0.36 |
|  | Intervention:Anxiety | 1 | 1.28 | 0.264 | 0.018 | 0.21 |
|  | Residuals | 43 |  |  |  |  |
| **Suppression** | Baseline | 1 | 36.19 | 0 | 0.445 | 1.00 |
|  | Group | 1 | 1.64 | 0.208 | 0.02 | 0.25 |
|  | Anxiety | 1 | 0.32 | 0.574 | 0.004 | 0.09 |
|  | Intervention:Anxiety | 1 | 0.15 | 0.703 | 0.002 | 0.07 |
|  | Residuals | 43 |  |  |  |  |
| **TCAQ** | Baseline | 1 | 104.91 | 0 | 0.66 | 1.00 |
|  | Group | 1 | 0.64 | 0.427 | 0.004 | 0.13 |
|  | Anxiety | 1 | 9.81 | 0.003 | 0.062 | 0.88 |
|  | Intervention:Anxiety | 1 | 0.62 | 0.434 | 0.004 | 0.12 |
|  | Residuals | 43 |  |  |  |  |
| *Sleep* |  |  |  |  |  |  |
| **PSQI** | Baseline | 1 | 26.72 | 0 | 0.372 | 1.00 |
|  | Group | 1 | 0.14 | 0.713 | 0.002 | 0.07 |
|  | Anxiety | 1 | 1.88 | 0.177 | 0.026 | 0.28 |
|  | Intervention:Anxiety | 1 | 0.10 | 0.749 | 0.001 | 0.06 |
|  | Residuals | 43 |  |  |  |  |

**Supplementary Table 4**. Self-report measures adjusted means at follow-up with ANCOVA interaction

|  |  | High | Low | Intervention:  Anxiety  Interaction | |
| --- | --- | --- | --- | --- | --- |
|  |  | *M* (*SE*) | *M* (*SE*) | *P* | |
| *Anxiety* |  |  |  |  | |
| Trait | GOS | 41.57 (1.81) | 36.41 (1.88) |  | |
|  | Placebo | 45.56 (1.80) | 33.42 (1.83) | **0.023** | |
| State | GOS | 37.23 (1.90) | 29.54 (2.07) |  | |
|  | Placebo | 34.08 (2.02) | 31.40 (2.02) | 0.181 | |
| SAS-A | GOS | 44.17 (1.88) | 44.72 (1.98) |  | |
|  | Placebo | 45.40 (1.77) | 42.74 (1.84) | 0.377 | |
| *Mood* |  |  |  |  | |
| MFQ | GOS | 5.75 (1.01) | 3.38 (1.09) |  | |
|  | Placebo | 6.09 (1.07) | 2.31 (1.08) | 0.475 |  |
| BDI-II | GOS | 9.46 (1.30) | 7.88 (1.40) |  | |
|  | Placebo | 9.07 (1.38) | 5.07 (1.39) | 0.354 | |
| *Emotion Regulation* | |  |  |  | |
| Reappraisal (ERQ-CA) | GOS | 20.72 (.090) | 21.10 (0.93) |  | |
|  | Placebo | 20.22 (0.86) | 22.62 (0.90) | 0.264 | |
| Suppression (ERQ-CA) | GOS | 11.57 (0.79) | 11.44 (0.83) |  | |
|  | Placebo | 10.85 (0.76) | 10.11 (0.79) | 0.703 | |
| TCAQ | GOS | 77.92 (2.54) | 86.33 (2.79) |  | |
|  | Placebo | 73.60 (2.61) | 85.79 (2.63) | 0.434 | |
| *Sleep* |  |  |  |  | |
| PSQI | GOS | 5.43 (0.63) | 4.71 (0.68) |  | |
|  | Placebo | 5.95 (0.64) | 4.81 (0.63) | 0.749 | |
|  |  |  |  |  | |
|  |  |  |  |  | |
|  |  |  |  |  | |
|  |  |  |  |  | |


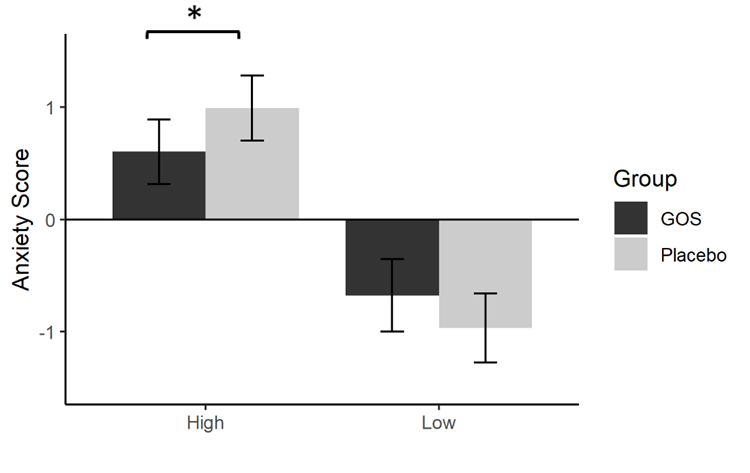


**Supplementary Figure 1.** *Trait anxiety scores at follow-up (plotted as z-scores, error bars are SE). Scores are reduced in the high anxious (x-axis) GOS group compared to the high anxious placebo group. * p* = .062.

**Supplementary Table 5.** Descriptive statistics for bacterial diversity by intervention and anxiety group.

|  | Baseline | Follow up |
| --- | --- | --- |
|  | *Mean* (*SD*) | *Mean* (*SD*) |
| ***Intervention*** |  |  |
| GOS | 4.84 (0.84) | 4.80 (0.80) |
| Placebo | 4.83 (0.82) | 5.09 (0.60) |

**References**

1 Spielberger, C. D., Gorsuch, R. L., Lushene, R., Vagg, P. R. & Jacobs, G. A. (Mind Garden Inc. 855 Oak Grove Ave., Suite 215, Menlo Park, CA 94025, 1970).

2 La Greca, A. M. (University of Miami, Miami, Florida, 1999).

3 Angold, A. *et al.* The development of a short questionnaire for use in epidemiological studies of depression in children and adolescents. *Journal of Methods in Psychiatric Research* **5**, 237-249 (1995).

4 Beck, A. T., Steer, R. A. & Brown, G. K. 38 (The Psychological Corporation, 555 Academic Court, San Antonio, TX 78204-2498, 1999).

5 Gullone, E. & Taffe, J. The Emotion Regulation Questionnaire for Children and Adolescents (ERQ-CA): a psychometric evaluation. *Psychol Assess* **24**, 409-417, doi:10.1037/a0025777 (2012).

6 Luciano, J. V., Algarabel, S., Tomás, J. M. & Martínez, J. L. Development and validation of the thought control ability questionnaire. *Personality and Individual Differences* **38**, 997-1008, doi:10.1016/j.paid.2004.06.020 (2005).

7 Buysse, D. J., Reynolds, C. F., 3rd, Monk, T. H., Berman, S. R. & Kupfer, D. J. The Pittsburgh Sleep Quality Index: a new instrument for psychiatric practice and research. *Psychiatry Res* **28**, 193-213 (1989).

8 Edgar, R. C. Search and clustering orders of magnitude faster than BLAST. *Bioinformatics* **26**, 2460-2461, doi:10.1093/bioinformatics/btq461 (2010).

9 Wang, Q., Garrity, G. M., Tiedje, J. M. & Cole, J. R. Naive Bayesian classifier for rapid assignment of rRNA sequences into the new bacterial taxonomy. *Appl Environ Microbiol* **73**, 5261-5267, doi:10.1128/AEM.00062-07 (2007).
